# A Phenomenological Model of the Electrically Stimulated Auditory Nerve Fiber: Temporal and Biphasic Response Properties

**DOI:** 10.3389/fncom.2016.00008

**Published:** 2016-02-08

**Authors:** Colin D. F. Horne, Christian J. Sumner, Bernhard U. Seeber

**Affiliations:** ^1^Medical Research Council Institute of Hearing Research, University ParkNottingham, UK; ^2^Audio Information Processing, Department of Electrical and Computer Engineering, Technische Universität MünchenMunich, Germany

**Keywords:** auditory nerve, electrical stimulation, cochlear implant, spike timing, computational modeling

## Abstract

We present a phenomenological model of electrically stimulated auditory nerve fibers (ANFs). The model reproduces the probabilistic and temporal properties of the ANF response to both monophasic and biphasic stimuli, in isolation. The main contribution of the model lies in its ability to reproduce statistics of the ANF response (mean latency, jitter, and firing probability) under both monophasic and cathodic-anodic biphasic stimulation, without changing the model's parameters. The response statistics of the model depend on stimulus level and duration of the stimulating pulse, reproducing trends observed in the ANF. In the case of biphasic stimulation, the model reproduces the effects of pseudomonophasic pulse shapes and also the dependence on the interphase gap (IPG) of the stimulus pulse, an effect that is quantitatively reproduced. The model is fitted to ANF data using a procedure that uniquely determines each model parameter. It is thus possible to rapidly parameterize a large population of neurons to reproduce a given set of response statistic distributions. Our work extends the stochastic leaky integrate and fire (SLIF) neuron, a well-studied phenomenological model of the electrically stimulated neuron. We extend the SLIF neuron so as to produce a realistic latency distribution by delaying the moment of spiking. During this delay, spiking may be abolished by anodic current. By this means, the probability of the model neuron responding to a stimulus is reduced when a trailing phase of opposite polarity is introduced. By introducing a minimum wait period that must elapse before a spike may be emitted, the model is able to reproduce the differences in the threshold level observed in the ANF for monophasic and biphasic stimuli. Thus, the ANF response to a large variety of pulse shapes are reproduced correctly by this model.

## Introduction

Cochlear implants restore the perception of sound to deafened individuals. The speech processor maps acoustic waveforms to trains of electrical pulses at each electrode of an array inserted in the cochlea, which directly stimulate auditory nerve fibers (ANFs). Cochlear implantees often achieve high levels of speech understanding in quiet single-talker situations. However, they are significantly disadvantaged compared with normal hearing and even moderately hearing impaired listeners in complex and noisy acoustic environments (e.g., Cullington and Zeng, 2008; Wilson and Dorman, 2008; Kerber and Seeber, 2012).

To restore hearing, the cochlear implant must convey sufficient information about the acoustic scene to the central auditory system. It is less clear how much information would be required to restore “normal” levels of functionality and how to encode information optimally given the limitations of the electrode-nerve interface. It is clear that only some of the normal acoustic “cues” are available with contemporary cochlear implants. In the predominant coding strategy individual electrodes carry discrete current pulses at a fixed rate, and the amplitude of the current is modulated according to the extracted envelope of sound in a fixed frequency range (Continuous interleaved sampling strategy, CIS, Wilson et al., 1991). Fine temporal information is removed by the speech processor and not coded in pulse timings. This information is known to be important for the perception of pitch and sound localization, both of which strongly influence the process of forming discrete perceptual acoustic objects from a mixture in normal hearing.

How the information should be encoded, given the limitations of the devices, is a difficult question. One potential method for manipulating ANF responses is via the shapes of electrical pulses. When stimulated with an electrical current pulse, the ANF may elicit an action potential after a stochastic delay. The shape of the stimulating pulse affects the probability of the ANF eliciting an action potential in response to it, and the temporal distribution of the action potential, if elicited. Further, temporally-separated pulses may interact within short time windows, blurring the distinction between individual pulses. Current implants use only biphasic pulses with identically shaped phases. Transforming a cathodic monophasic pulse into a cathodic-anodic biphasic pulse by introducing a trailing, anodic phase is necessary to achieve charge balance, a prerequisite for long-term use in patients. The additional anodic phase decreases the probability of the stimulus evoking an action potential in the ANF, but less so if a delay, or interphase gap (IPG), is introduced between the two opposite-polarity phases (Shepherd and Javel, 1999). The requirement to charge balance can be met by a wide range of pulse-shapes.

Future stimulation strategies might manipulate pulse-shape to improve information transmission or to reduce power consumption. For example, pseudomonophasic pulses with a short cathodic stimulating phase followed by a longer anodic phase of lower, charge-balanced amplitude are more efficient for stimulation and yield a larger dynamic range than biphasic pulses (Macherey et al., 2006), phase duration and interphase-gap have a pronounced effect on loudness (Carlyon et al., 2005), and the polarity order of multiphasic pulses can alter perceived pitch (van Wieringen et al., 2008; Carlyon et al., 2013). Further, strategies with novel pulse shapes have the potential to control the negative impact of current spread, e.g., by varying interphase gap, phase duration and the relative amplitude of the second phase, thereby changing firing probability of neurons in a larger region around the electrode. Moreover, when attempting to code fine temporal information, e.g., binaural cues needed for sound localization, the exact timing of pulses in the auditory nerve becomes crucial.

It is hard to evaluate the effectiveness of a stimulation strategy. It would be useful to observe the responses of the individual fibers of the stimulated auditory nerve. However, recording from single nerve fibers is an invasive procedure that is not possible in patients. Measures like neural response telemetry cannot be used with regular stimulation strategies and auditory brainstem responses cannot give insight into the responses of individual nerve fibers. Computational neural models can help predict the neural response when stimulating with changing pulse patterns and shapes, and hence help with the development of future stimulation strategies. Models could be used to find a stimulation pattern for which the neural response matches a target response as closely as possible, or to maximize information transmission. To this end, we have developed a model that simulates the auditory nerve fiber response to an electrical stimulus, which is sensitive to pulse shape parameters.

To be useful for developing stimulation strategies that manipulate pulse shape, a model must be capable of realistically responding to a stimulus pulse of complex shape, with varying phase durations and interphase gaps. One method by which to achieve this is to directly model the biophysics of the neuron. Biophysical models have been developed which are successful in reproducing the response characteristics of the ANF (e.g., Rubinstein, 1995; Cartee, 2000; Rattay et al., 2001; Negm and Bruce, 2008; Woo et al., 2010). However, while they have previously been used to study the responses of large populations of ANFs (e.g., Imennov and Rubinstein, 2009), they are difficult to use: the parameter-space of a biophysical model is vast and the individual parameters affect the response of the neuron in complex ways. There has been no procedure published for systematically parameterizing a biophysical model to reproduce a desired set of response statistics.

Phenomenological models provide an alternative to biophysical models. Phenomenological models reproduce only the statistics of the response, without explicitly modeling the biophysics of the ANF. By doing so, the parameter-space is reduced and it is possible to directly and independently control individual response characteristics via the model parameters. A variety of phenomenological models have been developed to reproduce the responses to sensory inputs or synaptic input (e.g., McGregor, 1987; Gerstner and Kistler, 2002; Izekevich, 2003). They rely on the fact that many of the complexities of their behavior, such as spike generation, are stereotypical. Perhaps the most commonly used model is the leaky-integrate-and-fire (LIF) model (for a review see Gerstner and Kistler, 2002). This has linear subthreshold filtering of the inputs, a fixed spike threshold and dispenses with all the dynamics of the spike generation.

Phenomenological models have previously been used to model the electrically stimulated ANF (e.g., Bruce et al., 1999; Hamacher, 2004; Carlyon et al., 2005; Chen and Zhang, 2007; Macherey et al., 2007; Cohen, 2009a,b,c,d; Chen, 2012; Goldwyn et al., 2012). The required constraints on these models are different to those of other domains. Whereas models with deterministic intrinsic properties (e.g., Rothman and Manis, 2003; Laudanski et al., 2010) are adequate to explain the responses to intracellular current injection or synaptic input in the auditory brainstem, and many other central neurons, modeling ANF responses to electrical stimulation requires a stochastic model. Models that incorporate noise into the firing threshold (Bruce et al., 1999; Gerstner and Kistler, 2002) allow for realistic firing probabilities for some stimulation protocols. However, the latency of firing does not emerge naturally in these models, which requires still further sources of stochasticity (Hamacher, 2004), and neither does the sensitivity to pulse shape.

The focus of this study has been to develop a model capable of reproducing the statistics of the ANF's response to both monophasic and biphasic stimuli of arbitrary amplitude, phase duration and interphase gaps. The model presented is the first phenomenological model to respond directly to a range of current pulse shapes and reproduce the effect that an immediate or delayed trailing, anodic phase has on the probability of a cathodic stimulus evoking an action potential in the ANF. The model is computationally efficient and easily parameterized, making it suitable for simulating the response of a large population of fibers.

Our model is based on the stochastic leaky integrate and fire (SLIF) neuron, a well-studied phenomenological model of the electrically stimulated neuron. The SLIF neuron discretizes the action potential as a single moment of spiking. In our model, the membrane potential of the ANF is modeled by processing the stimulus current with a leaky integrator. As in the SLIF neuron, excitation occurs when the membrane potential exceeds a stochastic threshold. Unlike the SLIF neuron, we add a delay between the moment at which the membrane potential exceeds the threshold and the moment at which the resulting spike is emitted. This emulates the delay in the generation of the action potential that is present in the ANF. Further, inspired by empirical observations, we allow the spike to be canceled if sufficient anodic stimulation occurs before the spike is emitted. By doing so, we are able to reproduce the effect of the interphase gap on the probability of a cathodic-anodic biphasic stimulus evoking an action potential in the ANF.

The description of our model is split into three sections. First, we introduce the existing SLIF neuron, describing its parameterization and summarizing its capabilities and limitations (Section Stochastic Leaky Integrate and Fire Neuron). We then extend the SLIF neuron to introduce a delay between the moment at which the membrane potential exceeds the threshold and the moment at which the resulting spike is elicited. This forms a self-contained model in itself, reproducing temporal properties of the ANF's response to a monophasic stimulus (Section Temporal Leaky Integrate and Fire Neuron). Finally, we further extend the model so that a spiking may be canceled by anodic current, comparing its results against those from cat ANFs (Section Biphasic Leaky Integrate and Fire Neuron).

## Stochastic leaky integrate and fire neuron

The stochastic leaky integrate-and-fire (SLIF) neuron provides a simple model of the electrically stimulated neuron. In the model, the neural membrane is considered to be a leaky integrator of current, with an associated membrane potential.

### Model description

The stimulus signal *I(t)* is processed by a leaky integrator to give *V(t)*, which can be interpreted as the membrane potential of the model neuron (Abbott and Kepler, 1990; Gerstner, 1995). The stimulus signal and the membrane potential are related by the ordinary differential equation

(1) $$\tau \frac{dV}{dt}=-RI-\;V,$$

where τ is the time constant of the neural membrane and *R* is its resistance, arbitrarily assumed to be 1Ω. A spike is generated at the moment *V(t)* first exceeds a threshold value θ, an event that we refer to as threshold crossing. Throughout the paper, we use *t*_0_ to denote the time of threshold crossing. In order to reproduce the stochastic properties of excitation, θ is a normally-distributed random variable with mean μ and standard deviation σ. Bruce et al. (1999) have demonstrated that this form of stochasticity provides for excellent fits for input-output functions of individual nerve fibers. Integrating Equation (1) we can obtain an expression for firing probability:

(2) $$P_{SLIF}=\Phi \left(\frac{-I\left[1-e^{-d/\tau }\right]-\mu }{\sigma }\right)$$

For a cathodic pulse of a duration, *d*, where Φ is the cumulative distribution function (CDF) of the Gaussian distribution. The model was implemented in Matlab, with leaky integration implemented via the *filter* function, with a sample rate of 1 MHz. Table 1 gives an overview of model parameters of the three models presented in this article and Table 2 summarizes all model variables.

**Table 1:** **Full set of model parameters and their values**.

**Parameter**	**Value**	**Models**	**Description**	**Cat ANF data**
μ	104.5 μV	SLIF, TLIF, BLIF	Mean of θ	Miller et al., 1999
σ	4.595 μV	SLIF, TLIF, BLIF	Standard deviation of θ	Miller et al., 1999
τ	248.4 μs	SLIF, TLIF, BLIF	Membrane time constant	van den Honert and Stypulkowski, 1984
*lat*(*p*): α_1_, α_2_, α_3_, α_4_	(see Figure 3A) 106 μV, 5.14 μV, 368 μS, 472 μS	TLIF, BLIF	Mean delay between start of action potential initiation period and spike observation, predicted from firing probability *p* as $lat\left(p\right)=\left(\frac{\alpha_{3}}{1+\exp\left(\alpha_{2}^{-1}\left(\mu +\sigma \phi^{-1}\left(p\right)-\alpha_{1}\right)\right)}\right)+\alpha_{4}$, where ϕ^−1^ is the quantile function of the standard normal distribution	Miller et al., 1999
*jit*(*p*): α_1_, α_2_, α_3_	(see Figure 3B) 109 μV, 3.24 μV, 136 μS	TLIF, BLIF	Standard deviation of the duration of the action potential initiation period, predicted from firing probability *p* as $jit\left(p\right)=\left(\frac{\alpha_{3}}{1+\exp\left(\alpha_{2}^{-1}\left(\mu +\sigma \phi^{-1}\left(p\right)-\alpha_{1}\right)\right)}\right)$, where ϕ^−1^ is the quantile function of the standard normal distribution	Miller et al., 1999
φ	37.81 μs	BLIF	Minimum delay between *t*_0_ and *t*_1_	Shepherd and Javel, 1999

Other than as specified in Figure 10, the given values were used to generate all the figures in this paper. The third column indicates which models use the given parameter, the value of which remains static between models. The fifth column cites the data against which the parameter was fitted.

**Table 2:** **Overview of model variables**.

**Variable**	**Models**	**Description**
*V*	SLIF, TLIF, BLIF	Membrane potential of neuron
θ	SLIF, TLIF, BLIF	Threshold against which *V* is compared
*t*_0_	SLIF, TLIF, BLIF	Time of threshold crossing; models the time at which the action potential initiation period begins
*t*_1_	TLIF, BLIF	Models the time at which the action potential initiation period ends
*t*_*spk*_	TLIF, BLIF	Models the time of action potential observation

The second column indicates which models use the given variable.

### Model response properties

The SLIF neuron has three parameters: μ, σ, and τ. In this section, we show how these can be uniquely determined to reproduce data from cat ANFs. We then outline the shortfalls of the SLIF neuron that will be addressed by the models presented in the remainder of the paper.

#### Excitation

As defined in this paper, the SLIF neuron is excited by negative, or cathodic, current. Positive, or anodic, current hyperpolarises the SLIF neuron, driving it further from excitation.

#### Input-output function

The input-output function of a neuron relates stimulus level to firing probability, for some stimulus pulse of fixed duration. It has been found that the input-output function of the ANF stimulated with a monophasic current pulse can be well approximated by the CDF of the Gaussian distribution (Dynes, 1996). The probability of a stimulus of current level *l* evoking an action potential is thus given by

(3) $$\Phi \left(\frac{l-m}{s}\right),$$

where Φ is the Gaussian CDF and *m* and *s* are the mean and standard deviation of the input-output function, respectively. The mean corresponds to the threshold level of the neuron—the level at which the neuron responds to the stimulus with a probability of 0.5. The standard deviation is a measure of the width of the input-output function, and thus, the dynamic range of the neuron. It is convenient to quantify the dynamic range as the ratio of the standard deviation and the mean (Verveen, 1961), giving *relative spread* (RS):

(4) $$RS=\;\frac{s}{m}.$$

The input-output function of the SLIF neuron (2) has the same form as Equation (3). Thus, by equating (2) and (3) and since *l* = −*I*,

(5) $$m=\;\frac{\mu }{1-\exp\left(-d/\tau \right)}$$

and

(6) $$s=\;\frac{\sigma }{1-\exp\left(-d/\tau \right)},$$

**Figure 1:**
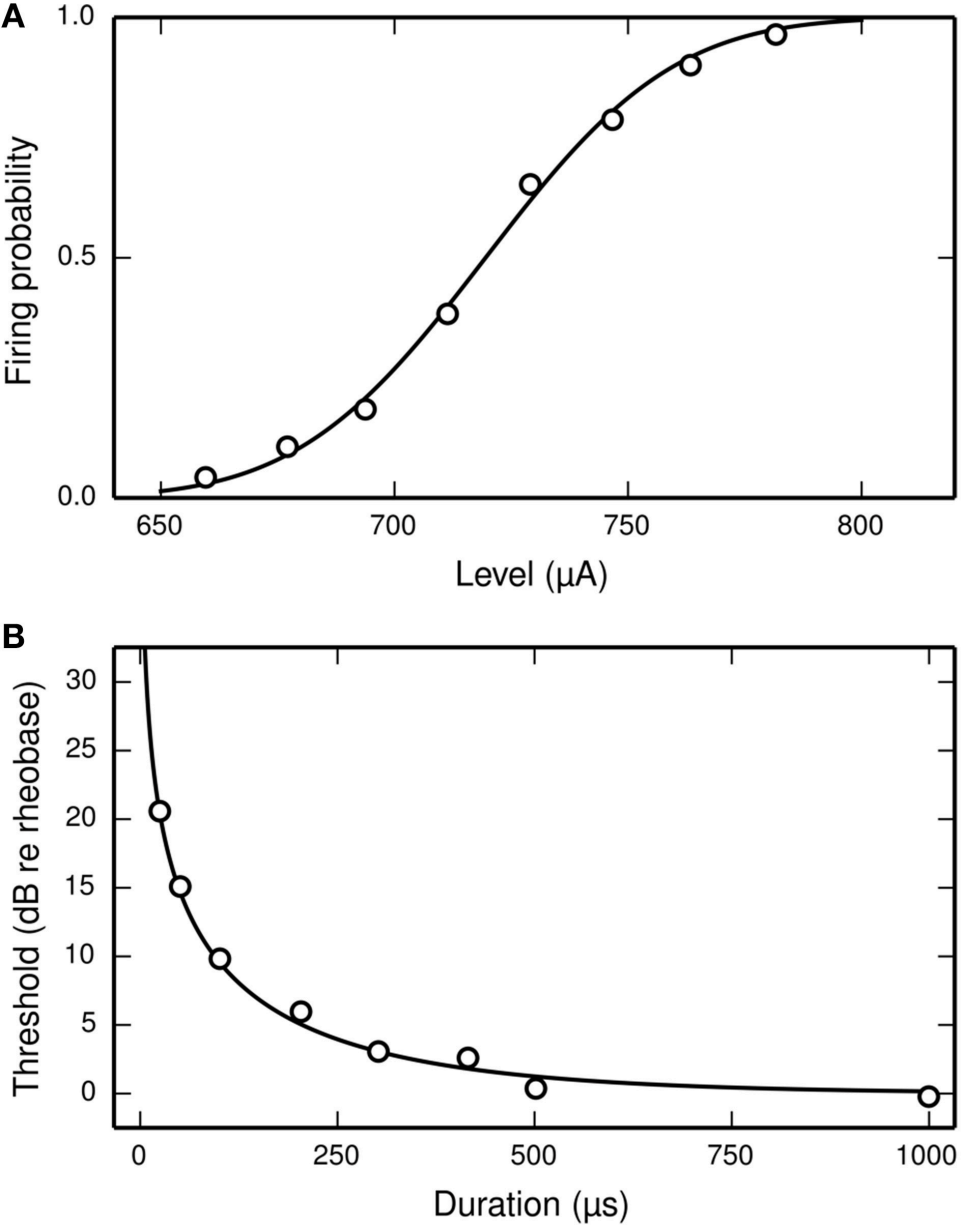
**The SLIF neuron may be parameterized to quantitatively reproduce input-output and strength-duration data**. **(A)** The input-output function of the SLIF neuron (solid line) fitted to data (open circles) from a cat ANF (Miller et al., 1999). The stimulus is a monophasic pulse (40 μs duration) presented in isolation. **(B)** The monophasic strength-duration function of the SLIF neuron (solid line) fitted to data (open circles) from a cat ANF (van den Honert and Stypulkowski, 1984).

Inverting these equations gives the values for the model parameters μ and σ that are needed for the SLIF neuron to reproduce the input-output function of an arbitrary ANF with threshold *m* and RS *s/m*. Increasing μ decreases the SLIF neuron's excitability and increasing σ increases its dynamic range. Figure 1A shows the input-output function of the SLIF neuron when parameterized to reproduce data for a cat ANF (Miller et al., 1999).

In the case of a monophasic stimulus, it has been hypothesized that the RS is a characteristic of the neuron and does not depend on stimulus duration (Verveen and Derksen, 1965). Like the real neuron, the RS of the SLIF neuron does not depend on stimulus duration.

#### Strength-duration function

The threshold level of a monophasic stimulus pulse depends on its duration, with greater durations incurring lower thresholds. The strength-duration function relates stimulus duration to threshold level and is often summarized by two measures: *rheobase* and *chronaxie*. As the stimulus duration increases, the threshold level reaches an asymptotic value—the rheobase. The stimulus duration that has a threshold level of twice the rheobase is the chronaxie. Measures of the chronaxie and strength-duration functions of cat ANFs were made by van den Honert and Stypulkowski (1984). They found that the threshold level *I*_*thr*_, when measured in amperes, was well predicted by the equation

(7) $$I_{thr}=\;\frac{I_{0}}{1-\exp(-kd)},$$

where *d* is the stimulus duration, in seconds, *I*_0_ is the rheobase, in amperes, and *log(2)*/*k* is the chronaxie, in seconds. The form of Equation (7) is consistent with other studies of neurons (e.g., Lapicque, 1907; Dean and Lawrence, 1985). The strength-duration function of the SLIF neuron has the same form, with *k* = 1∕τ (Hill, 1936). Inverting the equation gives the model parameter τ in terms of the chronaxie, allowing the model to reproduce the chronaxie of an arbitrary ANF. Figure 1B shows the strength-duration function of the SLIF neuron when parameterized to reproduce data from a cat ANF (den Honert and Stypulkowski, 1984).

#### Temporal response properties

**Figure 2:**
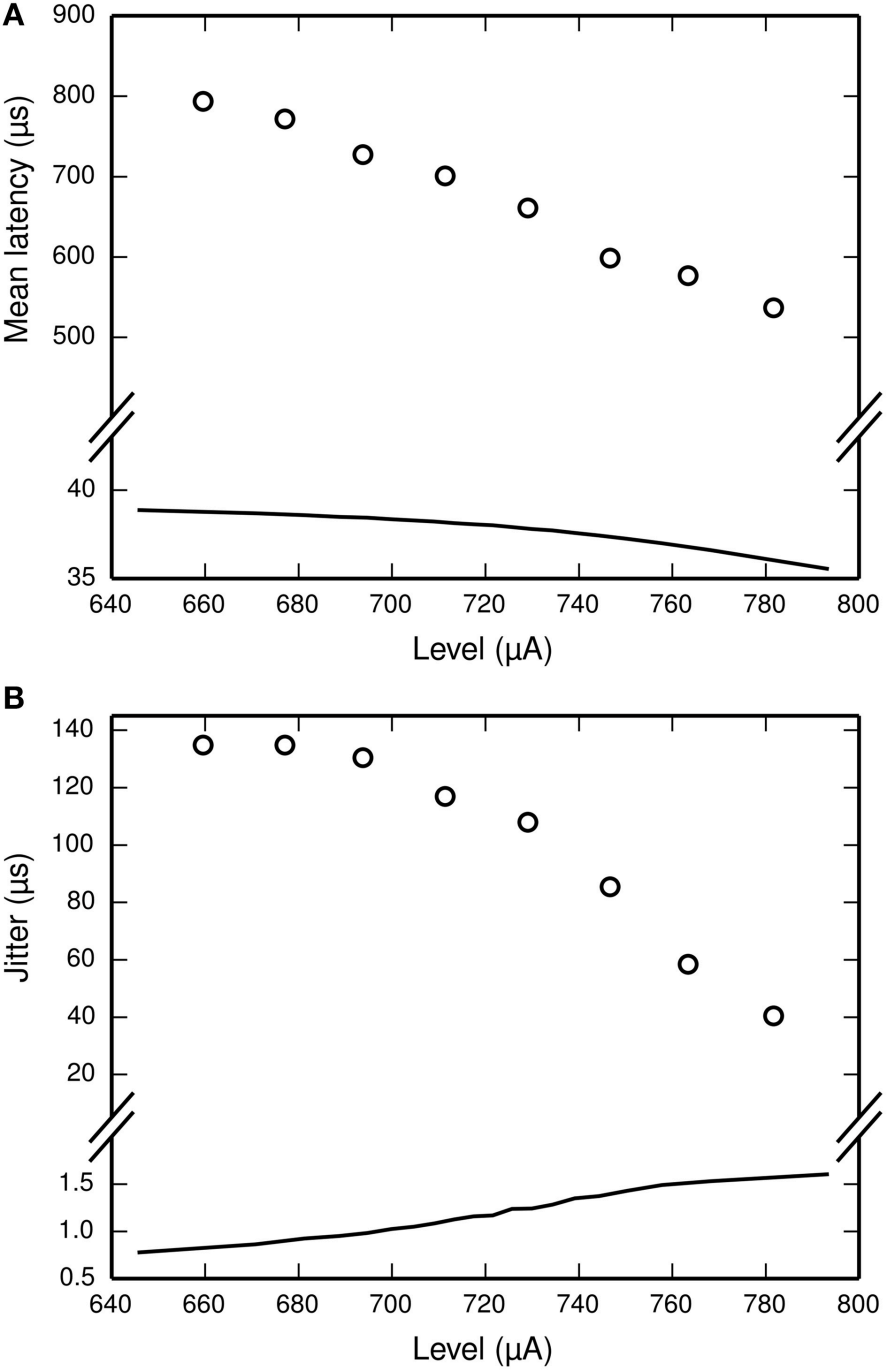
**The SLIF neuron does not reproduce the temporal response statistics of the ANF or their dependence on stimulus level. (A)** Mean latency, **(B)** Jitter of the responses to a monophasic stimulus (40 μs duration) for the SLIF neuron (solid lines) and a cat ANF (Miller et al., 1999; open circles). The stimulus levels span the dynamic range of the ANF. The cat ANF data used in Figure 1A and in this figure all come from the same ANF. Note the change in ordinate scale.

The latency of the ANF's response to a stimulus is defined as the delay between the onset of the stimulus and the observation of the action potential by the recoding electrode. It is stimulus-dependent and stochastic in nature. The jitter of the ANF's response is defined as the standard deviation of the latency. Figure 2 plots mean latency (Figure 2A) and jitter (Figure 2B) for a cat ANF's response to a brief (40 μs) monophasic stimulus (Miller et al., 1999). Increasing stimulus level reduces both the mean latency and the jitter of the response. Also plotted is the mean latency and jitter of the SLIF neuron under identical conditions. The SLIF neuron lacks the extent of temporal stochasticity that is observed in the ANF (jitter at threshold level is 1 μs for the model and 112 μs for the ANF). Further, the mean latency is under-predicted by the SLIF neuron (latency at threshold level is 38 μs for the model and 681 μs for the ANF) and does not show the dependence on stimulus level that is seen in the ANF. It is not possible to parameterize the SLIF neuron to reproduce these temporal response properties whilst simultaneously maintaining the input-output and strength-duration functions that have already been fitted to data from cat ANFs. These failings of the SLIF neuron have been noted previously (Hamacher, 2004; Fredelake and Hohmann, 2012; Goldwyn et al., 2012) and are addressed by our extension to the SLIF neuron in Section Temporal Leaky Integrate and Fire Neuron.

#### Biphasic response properties

The threshold level of a cathodic pulse is elevated by the inclusion of a trailing anodic phase, transforming it into a cathodic-anodic biphasic pulse (Gorman and Mortimer, 1983; Shepherd and Javel, 1999; Miller et al., 2001). As the IPG is increased, the threshold level tends toward that of the cathodic phase alone, reaching its asymptote after ~250 μs (Shepherd and Javel, 1999). The SLIF neuron is fundamentally unable to reproduce this increase in threshold level associated with cathodic-anodic biphasic stimulation. A threshold crossing, if one occurs, will always occur during the excitatory, cathodic current. If the threshold crossing occurs, then it cannot be undone by the trailing, anodic phase. If a threshold crossing does not occur during the leading cathodic phase, then it cannot occur during the trailing anodic phase. Thus, any trailing, anodic current present in a stimulus has no effect on the threshold level of that stimulus in the SLIF neuron.

### Summary

This section has introduced the SLIF neuron and shown that it may be analytically parameterized to reproduce the strength-duration and input-output functions of the ANF's response to a monophasic stimulus. The ease with which these important response statistics may be fitted to data makes the SLIF neuron an attractive candidate for modeling the response of the electrically stimulated ANF. However, we have also shown that the latency distribution of the SLIF neuron does not reproduce that of the ANF and that the SLIF neuron is unable to respond to a cathodic-anodic biphasic stimulus in a way that mimics the ANF.

## Temporal leaky integrate and fire neuron

In this section, we extend the SLIF neuron to reproduce the temporal properties of the ANF's response to a monophasic stimulus. We do so by introducing a stochastic delay between the time of threshold crossing and the time of spiking. The delay has no effect on the probability of the neuron responding to a stimulus, which is unchanged from that of the SLIF neuron. As such, the input-output and strength-duration functions of the SLIF neuron are preserved. We refer to the resulting model as the *temporal LIF* (TLIF) *neuron*.

### Model assumptions

The TLIF neuron makes a number of assumptions regarding how the ANF responds to the stimulus. We introduce these assumptions here, prior to providing a description of the model.

#### Predicting the latency distribution from the firing probability

We assume that the latency distribution of the ANF's response to a stimulus is well predicted by the probability of the stimulus obtaining a response. Thus, any changes in latency with the stimulus follows directly from the change in firing probability. We further assume that the latency distribution is well approximated by a Gaussian distribution.

#### The action potential initiation period

When a neuron is depolarized sufficiently to evoke an action potential, a delay occurs between the membrane being depolarized by the stimulus and the action potential being generated. During this delay, further stimulation can continue to affect the time at which the action potential is generated (van den Honert and Mortimer, 1979; Miller et al., 2001). We refer to this delay as the action potential initiation period. We assume that the duration of the action potential initiation period is stochastic and stimulus-dependent, with its variability equal to the variability of the spike timing that is observed by the recording electrode.

### Model description

Biophysically, the generation of an action potential is a continuous process occurring over a time course of hundreds of microseconds. Stimulation occurring during this time can continue to affect the latency distribution of the response (van den Honert and Mortimer, 1979). In the SLIF neuron, however, the action potential is considered a discrete moment of threshold crossing after which further stimulation has no effect. To allow the stimulus to continue to affect the latency distribution of the response *after* a threshold crossing, we discretize the action potential into three epochs. The first epoch, modeled by *t*_0_, is the moment at which the stimulus depolarizes the ANF, opening enough sodium channels to create a self-sustaining depolarization, so that an action potential will be generated in the absence of further stimulation. It signifies the start of the action potential initiation period. The second epoch, modeled by the variable *t*_1_, is the moment at which the action potential is irrevocably generated. This corresponds to the points at which sufficient sodium channels are open that action potential generation cannot be influenced by further stimulation. It signifies the end of the action potential initiation period, which is thus modeled by the interval [*t*_0_, *t*_1_]. After its initial generation, the action potential is conducted centrally by the axon until it is observed by the recording electrode, an event we refer to as action potential observation. The third epoch, modeled by the variable *t*_*spk*_, is the moment of action potential observation, when the extracellular potential at the recording electrode exceeds the threshold for spike detection.

As previously defined in Section Model Description, *t*_0_ is the time at which a threshold crossing occurs in the model. Upon a threshold crossing occurring in the TLIF neuron, a value is generated for the variable *t*_1_ such that the duration of the delay between *t*_0_ and *t*_1_ is exponentially distributed with a stimulus-dependent variance. A value is then generated for the variable *t*_*spk*_ so that the duration of the delay between *t*_0_ and *t*_*spk*_ has a normal distribution with a stimulus-dependent mean and variance. The variance is the same as for *t*_1_, though the mean is considerably larger so as to emulate the effects of axonal conductance on latency.

**Figure 3:**
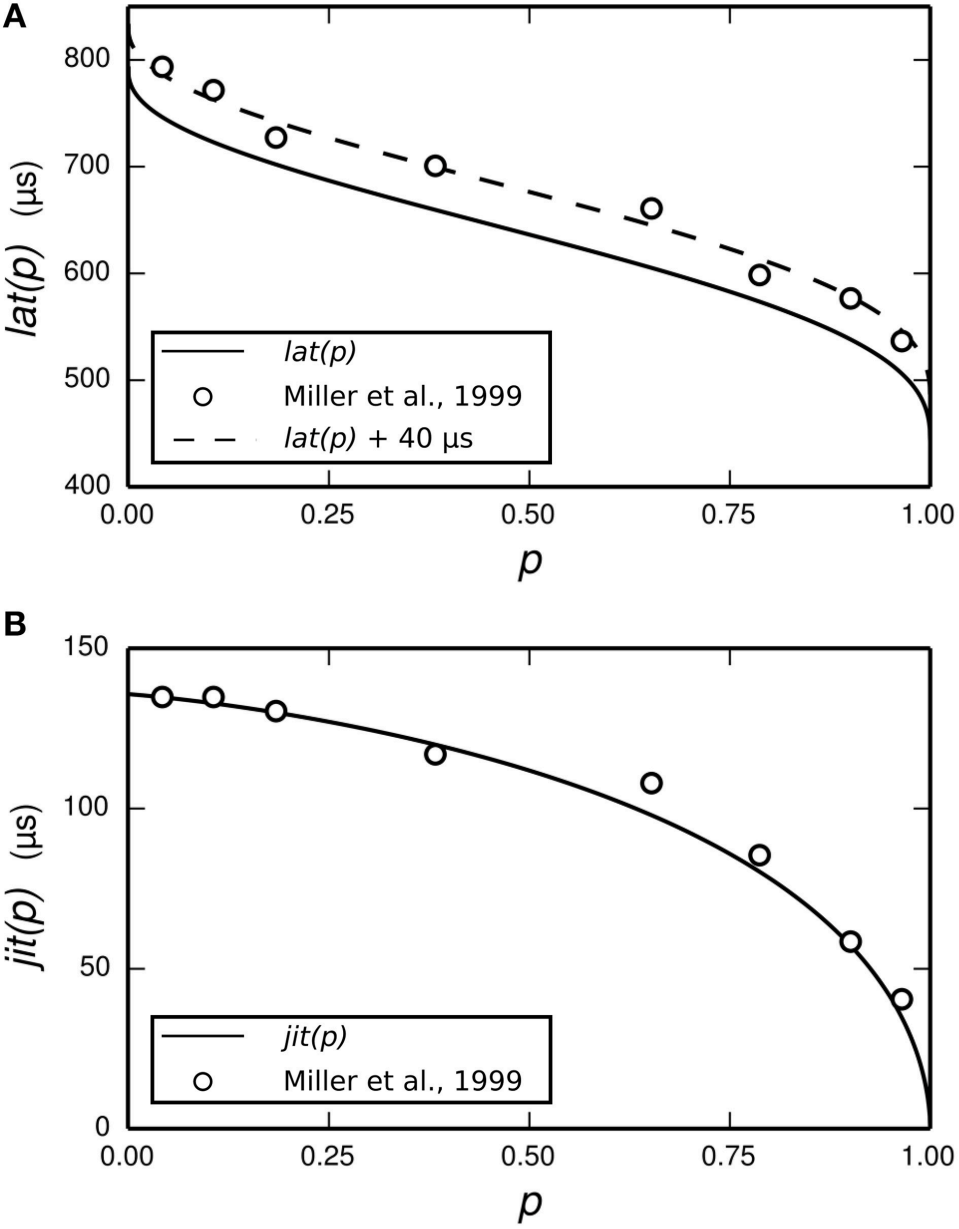
**Parameterization of the functions *lat* (solid line, A) and *jit* (solid line, B) from empirical data**. The function *lat* is fitted to the mean latency of an ANF's response to a 40 μs monophasic pulse (Miller et al., 1999; open circles, **A**). The action potential initiation period is assumed to begin at the time of stimulus cessation, and so *lat* is chosen to under-predict the data by 40 μs. We have also plotted *lat*+40 μs (dashed line, **A**) to better show the fit to data. The function *jit* is fitted to the jitter of the same ANF's response to the same stimulus (Miller et al., 1999; open circles, **B**).

The dependence of *t*_1_ and *t*_*spk*_ on the stimulus is achieved by use of two empirically-derived functions: *lat*(*p*) and *jit*(*p*), both of which are plotted in Figure 3. *lat*(*p*) predicts the mean delay between the start of the action potential initiation period and the moment of action potential observation in the ANF stimulated so as to evoke an action potential with probability *p*. *jit*(*p*) is similar, but predicts the standard deviation of the action potential initiation period. Both functions are obtained by interpolating empirical data (Miller et al., 1999) collected from a cat ANF. For the TLIF neuron to use these functions, it must predict the probability of the stimulus evoking an action potential in the ANF. The stimulus signal *I* may include an arbitrarily shaped current waveform extending back in time, but in the model the times *t*_1_ and *t*_*spk*_ are influenced only by the current occurring during the associated action potential initiation period, i.e., the interval [*t*_0_, *t*_1_]. The latency of the TLIF neuron is thus a function of the probability of the membrane potential exceeding the threshold during the interval [*t*_0_, *t*_1_], which may be different to the probability at *t*_0_. In the next section, we show how this probability is derived. In the sections that follow, we show how it is used to generate *t*_1_ and *t*_*spk*_.

#### Probability of the membrane potential exceeding the threshold during the interval [*t*_0_, *t*_1_]

The probability of the membrane potential exceeding the threshold during the interval [*t*_0_, *t*_1_] is given by the probability that *V(s)* > θ, where s is the time at which *V* is maximal within [*t*_0_, *t*_1_]. Because the lower bound of this interval, *t*_0_, is the time of threshold crossing, it must be the case that *V*(*t*_0_) is greater than *V*(*s*), for all values of *s* less than *t*_0_. Therefore, the lower bound of the interval is redundant and may be omitted without affecting the probability.

Let the probability of the membrane potential exceeding the threshold be:

(8) $$P_{TLIF}\left(t\right)=Pr(V_{peak}>\theta )=\Phi \left(\frac{V_{peak}-\mu }{\sigma }\right),$$

where *V*_*peak*_ is the maximum voltage within the interval [0, *t*], given by

(9) $$V_{peak}=\underset{s\in [0,\;t]}{\max}V(s),$$

and Φ is the Gaussian CDF (recalling that θ is a normally-distributed random variable with mean μ and standard deviation σ). The probability of the membrane potential exceeding the threshold during the interval [*t*_0_, *t*_1_] is then given by *P*_*TLIF*_*(t*_1_*)*.

#### Generation of *t*_1_, the end of the action potential initiation period

The variable *t*_1_ models the time at which the action potential initiation period ends. This section describes the algorithm used by the TLIF neuron to generate a value for *t*_1_ such that the delay between *t*_0_ and *t*_1_ has an exponential distribution with standard deviation that approximates the jitter of the ANF's response to the same stimulus. The algorithm maintains causality so that at simulation time *t*, the model only has access to the membrane potential up until time *t*.

From the time of threshold crossing onwards, the TLIF neuron generates time-varying estimates of *t*_1_. Let *Y* be an exponentially-distributed (unit rate constant) random variable. The estimate of *t*_1_ made at simulation time *t* is referred to as $\hat{t_{1}}\left(t\right)$ and given by

(10) $$\hat{t_{1}}\left(t\right)=t_{0}+Y\;jit\left(P_{TLIF}\left(t\right)\right).$$

The final value for *t*_1_ is the time at which *t* first reaches or exceed $\hat{t_{1}}\left(t\right)$, at which point the action potential initiation period has already come to an end (at time $\hat{t_{1}}\left(t\right)$) and so its end time is no longer in flux. The time *t*_1_ is thus a fixed point of $\hat{t_{1}}$; that is, $\hat{t_{1}}\left(t_{1}\right)=t_{1}.$ Because *jit*(*P*_*TLIF*_(*t*)) is non-negative and increases monotonically with *t*, the function $\hat{t_{1}}$ has a single, unique, fixed point: *t*_1_. The fixed point was found by the bisection method.

**Figure 4:**
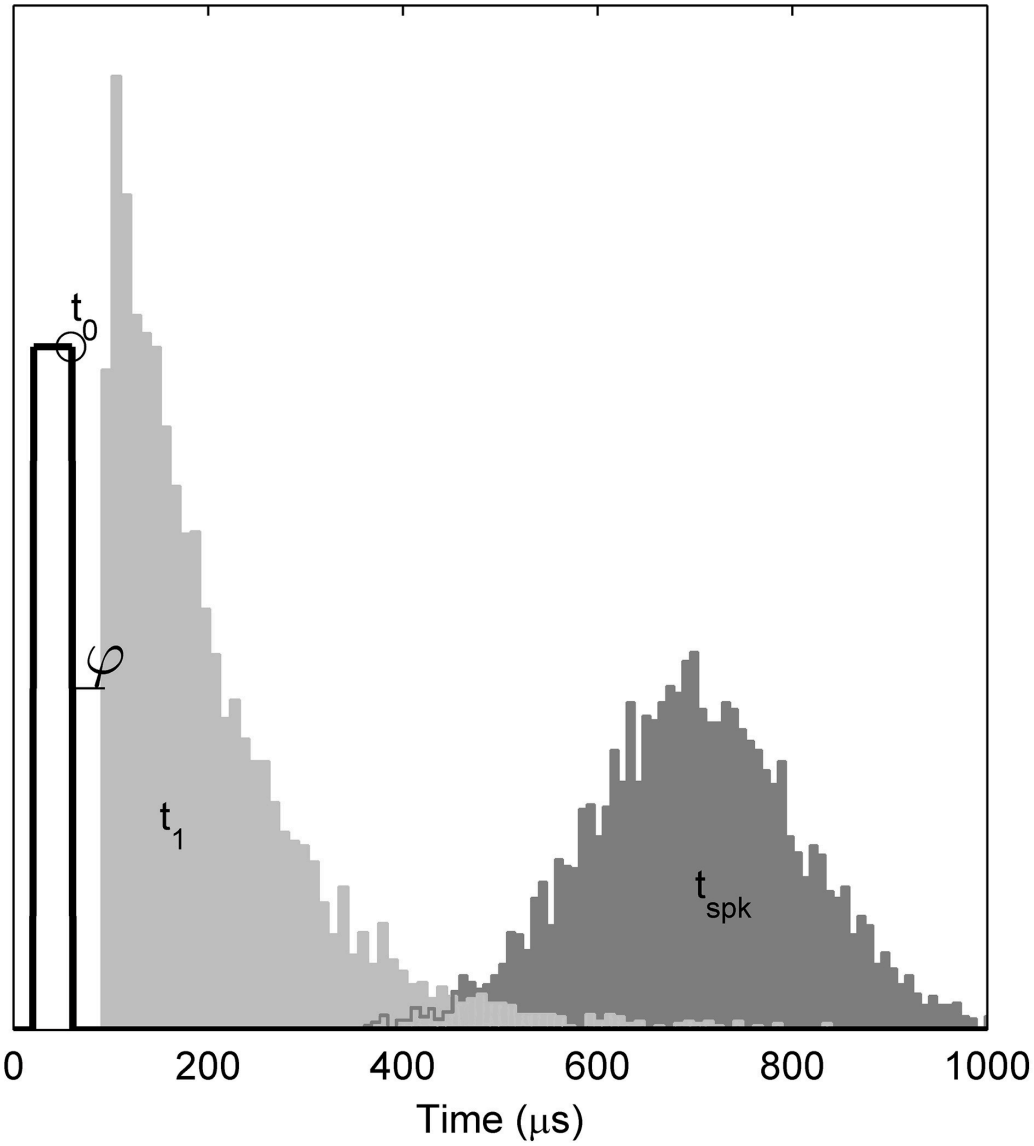
**Temporal distributions of the TLIF neuron's response to a threshold-level monophasic stimulus (40 μs duration, 5000 presentations)**. Histograms show the distributions taken by model variables *t*_1_ and *t*_*spk*_. Also marked is the mean value of t_0_ (open circle), the stimulus pulse (solid line; arbitrary ordinate units). The model parameter φ, which delays *t*_1_, is zero in the TLIF model but is shown here for consistency with the BLIF model. The variability of *t*_0_ is small (1.2 μs) and omitted from the plot for clarity. If the model were to be further stimulated during or before the light gray region (*t*_1_), the distribution of *t*_*spk*_ would be affected. However, if stimulated after the light gray region, the distribution of *t*_*spk*_ would be unaffected.

Empirically, when responding to a single, brief (< 100 ms) stimulus presented at a level sufficient to evoke a response with probability *p*, the TLIF neuron generates values of *t*_1_ with a standard deviation close to *jit*(*p*), while also ensuring that any stimulus current occurring after *t*_1_ has no effect on the value of *t*_1_. As an example of the algorithm, Figure 4 plots the distribution of values taken by *t*_1_ in response to a short-duration monophasic stimulus.

#### Generation of *t*_*spk*_, the time of action potential observation

After the action potential initiation period ends at time *t*_1_, the TLIF neuron generates *t*_*spk*_, modeling the time of action potential observation, taking into account the delay associated with axonal conductance. The time *t*_*spk*_ is generated so that, across repeated trails to the same stimulus, the delay between *t*_0_ and *t*_*spk*_ is normally distributed with mean and standard deviation given by *lat*(*p*) and *jit*(*p*), respectively, where *p* is the probability that the membrane potential exceeds threshold during the interval [*t*_0_, *t*_1_]. Formally,

(11) $$t_{spk}=t_{0}+X\;jit\left(p\right)+lat\left(p\right),$$

where *p* = *P*_*TLIF*_(*t*_1_) and *X* is a standard normal random variable.

#### Relationships between model variables

**Figure 5:**
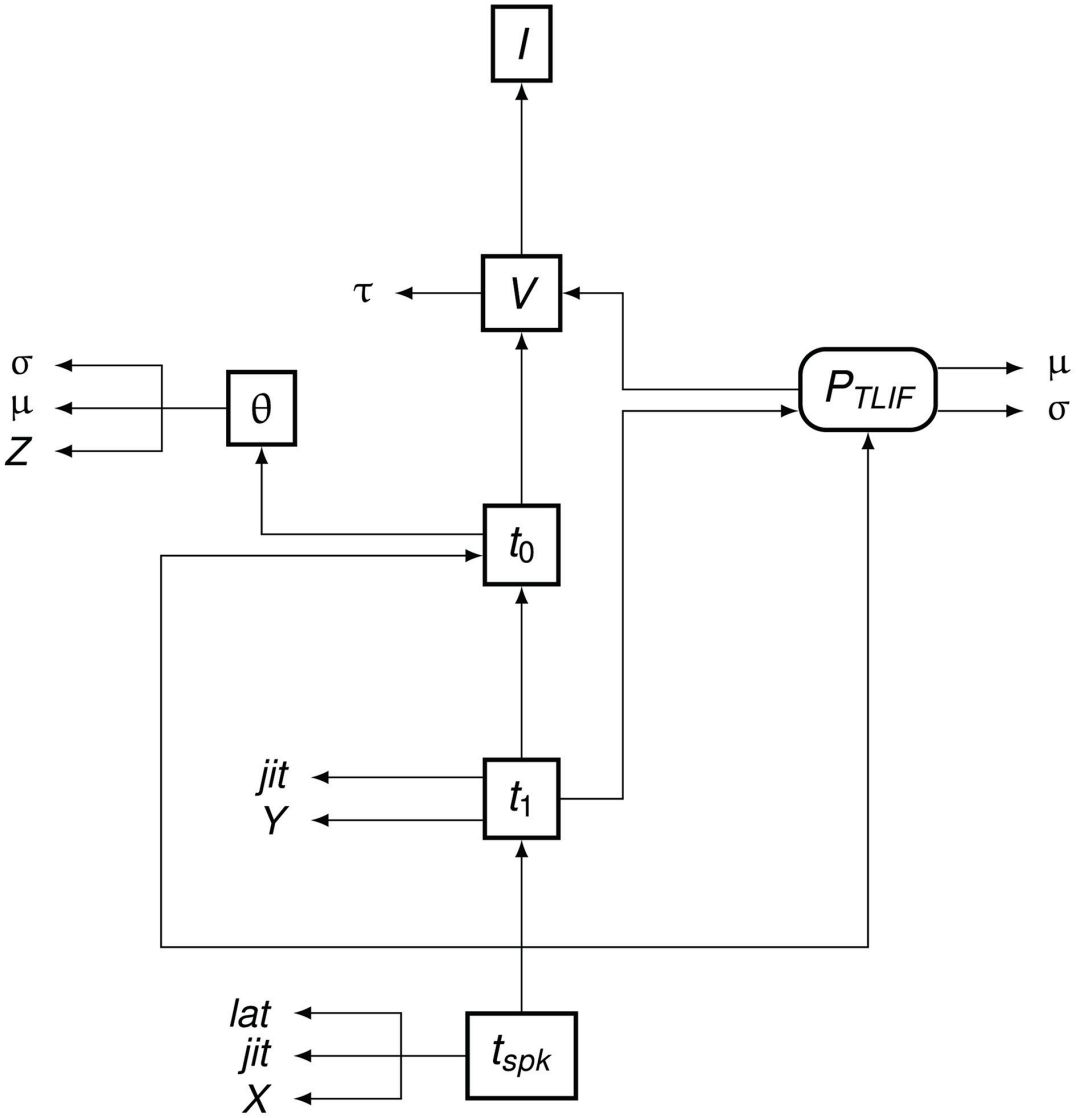
**Data dependency in the TLIF neuron**. The entities are the functions, variables, and parameters of the model. Each entity points to those on which its value depends. For example, *t*_*spk*_ depends on *t*_1_, *t*_0_ and *P*_*TLIF*_. Boxed Boxes entities with sharp edges are model variables and boxed entities with rounded edges are functions. The unboxed entities *X, Y*, and *Z* are random variables. The other unboxed entities are model parameters.

To summarize the flow of information in the TLIF neuron, Figure 5 shows how the different model parameters, variables, and functions are related to one another. The input to the model is the current signal *I* and the final output is the time of action potential observation, *t*_*spk*_.

### Model response properties

The TLIF neuron responds to a stimulus with the same probability as the SLIF neuron: upon a threshold crossing, both models are guaranteed to emit a spike. Thus, the input-output and strength-duration functions of the SLIF neuron are preserved in the TLIF neuron, without the need to change any of the SLIF neuron's parameters. However, the time *t*_*spk*_ of the spiking in the TLIF neuron is changed so as to better reflect the latencies observed empirically in the ANF.

**Figure 6:**
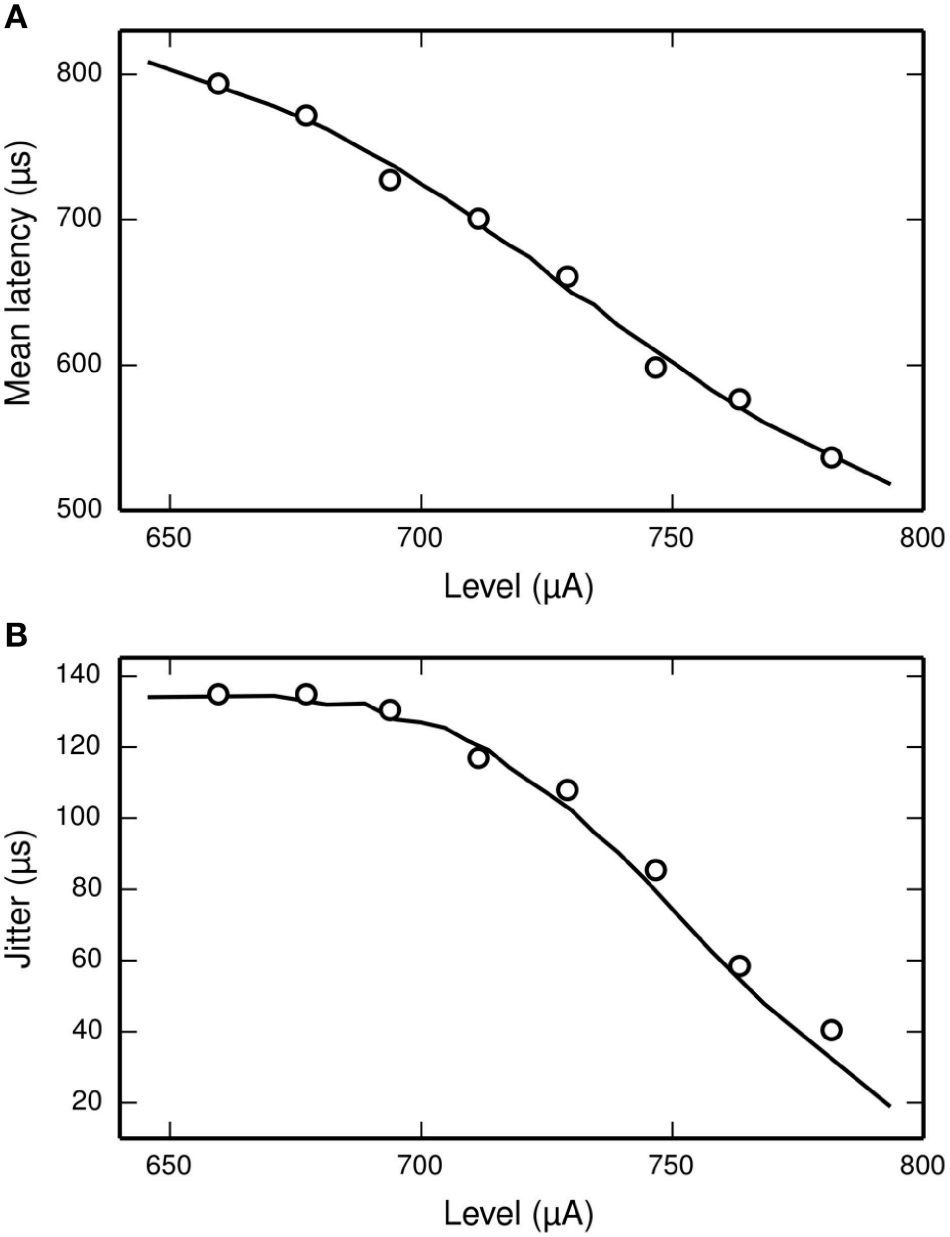
**The TLIF neuron reproduces the temporal response statistics of the ANF and their dependence on stimulus level**. **(A)** Mean latency (solid line). **(B)** Jitter (solid line) of the TLIF neuron's responses to a monophasic stimulus (40 μs duration) presented at levels spanning the neuron's dynamic range. The mean latency and jitter data used in Figure 2 are also plotted (Miller et al., 1999; open circles).

Miller et al. (1999) recorded the mean latency and jitter of the responses of an ANF to a monophasic stimulus presented at a range of stimulus levels spanning the dynamic range of the ANF. Their results are plotted in Figure 6, along with the corresponding results from the TLIF neuron. The TLIF neuron is able to quantitatively reproduce the mean latency and jitter of the ANF at all stimulus levels. Regardless of stimulus level, the latency distribution of the TLIF neuron is well approximated by a Gaussian distribution, a close approximation to that of the cat ANF (Javel and Shepherd, 2000).

### Summary

In Section Temporal Leaky Integrate and Fire Neuron, we extended the SLIF neuron so as to model the action potential initiation period of the ANF, resulting in the TLIF neuron. Stimulus current occurring during the action potential initiation period continues to affect the latency of the spike, although the spike's probability is unaffected. The TLIF neuron predicts the latency distribution with which the ANF would have responded to the stimulus by using the probability that the membrane potential exceeds threshold during the action potential initiation period. By doing so, the TLIF neuron is able to quantitatively reproduce the mean and standard deviation of the latency of the ANF's response to a monophasic stimulus, and the dependence of both on stimulus level.

## Biphasic leaky integrate and fire neuron

Under cathodic-anodic biphasic stimulation, the SLIF neuron is not affected by the trailing, anodic phase of the stimulus because any threshold crossing will always occur before the onset of the anodic phase. The TLIF neuron models the action potential initiation period by introducing a delay after threshold crossing during which stimulation may continue to influence the time of the generated spike. However, the probability of a spike being emitted is unchanged during this delay, and so the TLIF neuron suffers the same shortcoming in responding to a cathodic-anodic biphasic stimulus as the SLIF neuron. In this section, we further extend the model so that anodic current is able to affect the probability of the spike being generated. By doing so, we are able to reproduce the response statistics of a cathodic-anodic biphasic stimulus, without disrupting the monophasic response statistics. We refer to the final model as the *biphasic LIF* (BLIF) *neuron*.

### Model assumptions

We assume that spiking may be canceled by anodic current up until the end of the action potential initiation period at time *t*_1_. The ability for anodic currents to abolish an action potential that would have otherwise been generated has been observed experimentally in animal preparations (Tasaki, 1956; van den Honert and Mortimer, 1979; Weitz et al., 2011).

### Model description

The BLIF neuron is similar to the TLIF neuron: the time course of the action potential is modeled by the same three variables: *t*_0_, *t*_1_, and *t*_*spk*_. The equations used to generate the values of *t*_1_ and *t*_*spk*_ are parallels to those used by the TLIF neuron. The BLIF neuron differs from the TLIF neuron in that a spike may be canceled after a threshold crossing occurs. A spike is canceled if sufficient anodic charge is delivered during its associated action potential initiation period.

#### Generation of *t*_1_, the end of the action potential initiation period

The method by which the BLIF neuron generates the time *t*_1_ is similar to that of the TLIF neuron. However, in the BLIF neuron, it becomes useful to set a minimum possible duration for the action potential initiation period. To do so, we introduce a new model parameter: φ. The time *t*_1_ is now given by

(12) $$t_{1}=\max\left(\varphi ,\;t\right),$$

where *t* is such that $\hat{t_{1}}\left(t\right)=t$ and $\hat{t_{1}}$ is as defined previous. As we will show when analyzing the response properties of the BLIF neuron, increasing φ has the effect of decreasing the probability of a cathodic-anodic biphasic stimulus evoking a spike.

#### Spike cancelation

Given that a threshold crossing occurred at time *t*_0_ and the corresponding action potential initiation period ends at time *t*_1_, the impending spike is canceled if

(13) $$\int_{t_{0}}^{t}I\left(s\right)\;ds>0,\;for\;any\;t\in \left[t_{0},t_{1}\right].$$

That is, a spike is canceled if the total charge delivered during the action potential initiation period ever becomes positive (anodic). In the event of spike cancelation, the model terminates as though no threshold crossing occurred.

#### Probability of spiking in the BLIF neuron

To determining whether a spike will occur, the same methods as the TLIF model are used to generate *t*_1_, which depends on *P*_*TLIF*_. But whether a spike actually occurs also depends on the cancelation. This effectively modifies the observed probability of firing away from *P*_*TLIF*_. In the BLIF neuron, this is given by *P*_*BLIF*_(*t*_1_), the probability that the membrane potential exceeds threshold during the interval [*t*_0_, *t*_1_] and that the resulting spike is not subsequently canceled. This actual firing probability is required in order to infer the latency distribution with which to respond.

Let *T*_*Q*0_(*t*) denote the time at which the charge delivered by the stimulus since time *t* first becomes positive (anodic). Given that a threshold crossing occurs at time *t*_0_, the associated spike will be canceled if *T*_*Q*0_(*t*_0_) < *t*1. If *P*_*t*_1__(*t*; *t*_0_) denotes the probability that *t*_1_ occurs before time *t*, given *t*_0_, then the probability of spike survival (i.e., no cancelation) is *P*_*t*_1__(*T*_*Q*0_(*t*_0_) ; *t*_0_). In a single simulation, *t*_0_ has a fixed value. However, across repeated simulations to the same stimulus, *t*_0_ has a degree of variability which affects the probability of spike cancelation. Therefore, the probability derived by *P*_*t*_1__ must be integrated across time to account for the variability of *t*_0_. Formally,

(14) $$P_{BLIF}\left(t\right)=\int_{0}^{t}P_{TLIF}^{\prime }\left(s\right)\;P_{t_{1}}\left(T_{Q0}\left(s\right);s\right)\;ds,$$

where $P_{TLIF}^{\prime }$ denotes the derivative of *P*_*TLIF*_. The function *T*_*Q*0_(*t*) is defined as the smallest value of *s* for which $\int_{t}^{s}I\left(u\right)\;du<0$. If no such value for s exists, then *T*_*Q*0_(*t*) = ∞.

The general definition of *P*_*t*_1__ is given by

(15) $$P_{t_{1}}(t;t_{0})=\int_{t_{0}}^{t}\lambda (s)e^{-(s-t_{0})\lambda (s)}ds\;+\;\int_{t_{0}}^{t}(s-t_{0})e^{-(s-t_{0})\lambda (s)}\lambda^{\prime }(s)\;ds,$$

where λ(*s*) = 1∕*jit*(*s*) and λ′ is the derivative of λ. For computational efficiency, it is beneficial to find a closed form expression for *P*_t_1__. This is made possible by noting that λ is monotonically increasing, which allows for the simpler, computationally efficient formulation,

(16) $$P_{t_{1}}\left(t;s\right)=\{\begin{array}{cc} 1-exp\left(-\frac{t-s-\varphi }{jit\left(P_{TLIF}(t)\right)}\right), & t\geq s+\varphi \\ \;0, & t<s+\varphi \end{array}.$$

#### Generation of *t*_*spk*_, the time of spiking as observed by the recording electrode

The time *t*_*spk*_ at which the spike is observed by the recording electrode in the BLIF neuron is given by

(17) $$t_{spk}=t_{0}+X\;jit\left(p\right)+lat\left(p\right),$$

where *p* = *P*_*BLIF*_(*t*_1_) and *X* is a standard normal random variable. Equation (17) is identical to the corresponding Equation (11) in the TLIF neuron, but with the function *P*_*TLIF*_ replaced by the function *P*_*BLIF*_.

#### Relationships between model variables

**Figure 7:**
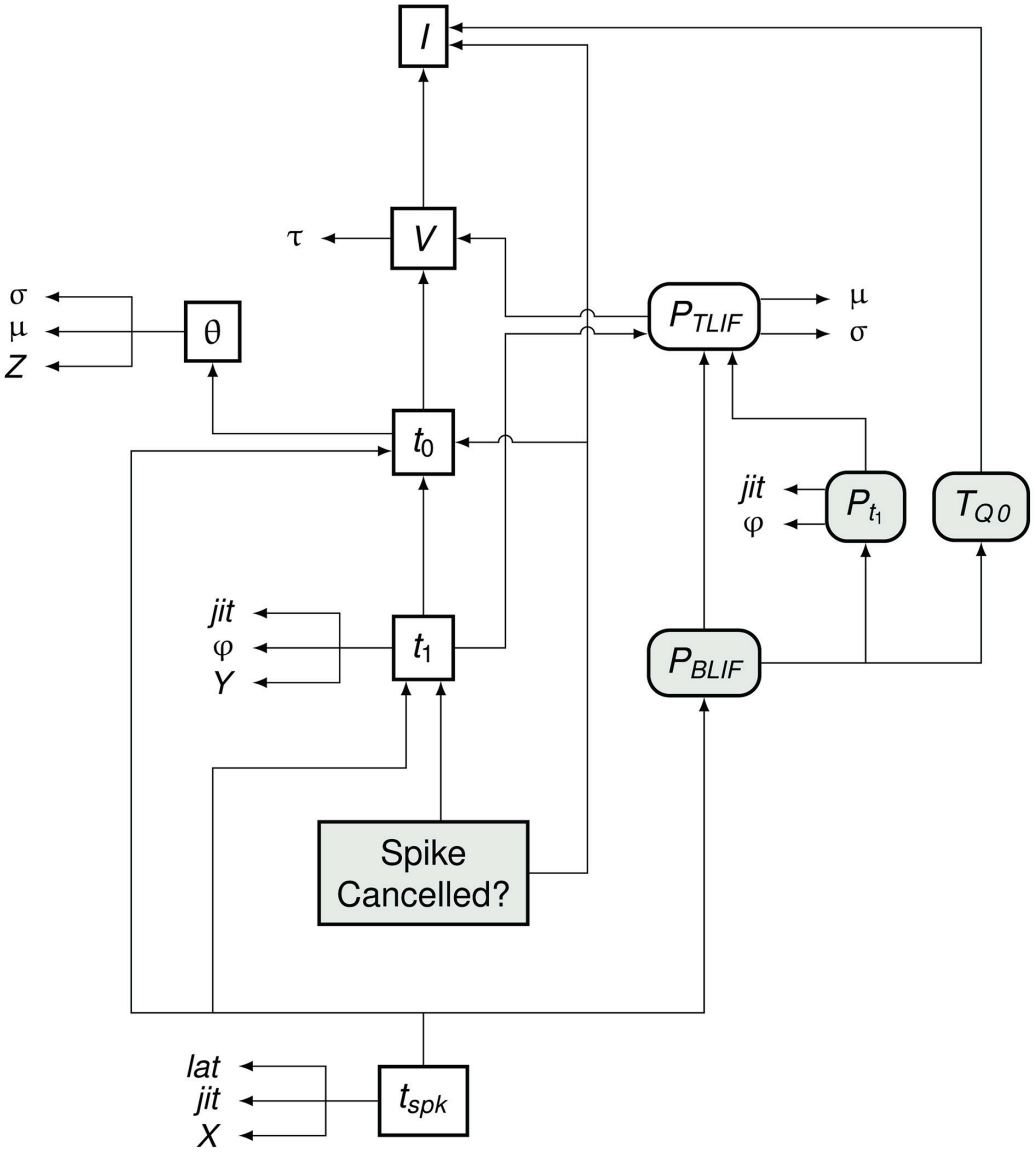
**Data dependency in the BLIF neuron**. The notation is as in Figure 4. Each entity points to those on which its value depends. For example, *t*_*spk*_ depends on *t*_1_, *t*_0_, and *P*_*BLIF*_. Entities which are present in the BLIF neuron but not the TLIF neuron are shaded gray. If the spike is to be canceled, then no value is generated for *t*_*spk*_. Comparing this diagram of the BLIF neuron against that of the TLIF neuron in Figure 5 shows the similarities between the two models.

Figure 7 summarizes the flow of information in the BLIF neuron. As in the TLIF neuron, the input to the model is the current signal *I*. However, unlike the TLIF neuron, the BLIF neuron has two outputs: the decision as to whether or not cancelation occurs and the time of action potential observation, *t*_*spk*_. In the case of cancelation, no value is generated for *t*_*spk*_.

### Model response properties

In the absence of anodic current, the probability of a stimulus evoking a spike in the BLIF neuron is unchanged from that in the TLIF neuron (without any anodic current, there is no possibility of a spike being canceled). Similarly, the latency distribution of the BLIF neuron's response to a monophasic stimulus is negligibly different to that of the TLIF neuron's (as will be demonstrated in Section Monophasic Response Latency). Therefore, with the exception of φ, which is specific to the BLIF neuron—the parameterization of the BLIF neuron remains as it was in the TLIF neuron and the BLIF neuron preserves the fitting of the input-output, strength-duration, latency-level, and jitter-level functions, in all cases reproducing empirically-derived data from cat ANFs.

### Monophasic response latency

If the stimulus signal *I* contains only excitatory, cathodic current and φ = 0, then the equations defining *t*_1_ and *t*_*spk*_ in the BLIF neuron become equivalent to those in the TLIF neuron. In the case of *t*_1_, this is a trivial consequence of Equation (12). The definitions of *t*_*spk*_ in the TLIF (11) and BLIF (17) neurons are the equivalent if *P*_*BLIF*_ = *P*_*TLIF*_. If *I* is a monophasic stimulus, then *T*_*Q*0_(*t*) = ∞ for all *t*, and so *P*_*t*_1__(*T*_*Q*0_(*t*) ; *t*) = 1 for all *t*. Equation (14) thus becomes

(18) $$P_{BLIF}\left(t\right)=\int_{0}^{t}P_{TLIF}^{\prime }\left(s\right)\;ds=P_{TLIF}\left(t\right),$$

and so the definitions of *t*_*spk*_ in the TLIF and BLIF neurons are equivalent if *I* is a monophasic stimulus.

#### Effect of φ on the latency distribution of the response to a monophasic stimulus

We now demonstrate that even when φ > 0, its effect on the final latency distribution (i.e., the distribution of *t*_*spk*_) is negligible. We analyzed the latency of the response of the BLIF neuron to a monophasic stimulus (40 μs pulse duration) when φ = 1 μs and φ = 60 μs, corresponding to biphasic/monophasic threshold differences of 0.95 and 11.7 dB respectively. For both values of φ, we recorded mean latency and jitter across 5 stimulus levels, evoking spikes with probabilities between 0.05 and 0.95. The difference in mean latency and jitter were noted for each stimulus level. The maximum difference in mean latency was 3.3 μs (2.6 × SE). The maximum difference in jitter was 1.5 μs (1.4 × SE). We therefore conclude that the effect of φ on the latency distribution of the response is negligible.

#### Effect of φ on biphasic threshold

**Figure 8:**
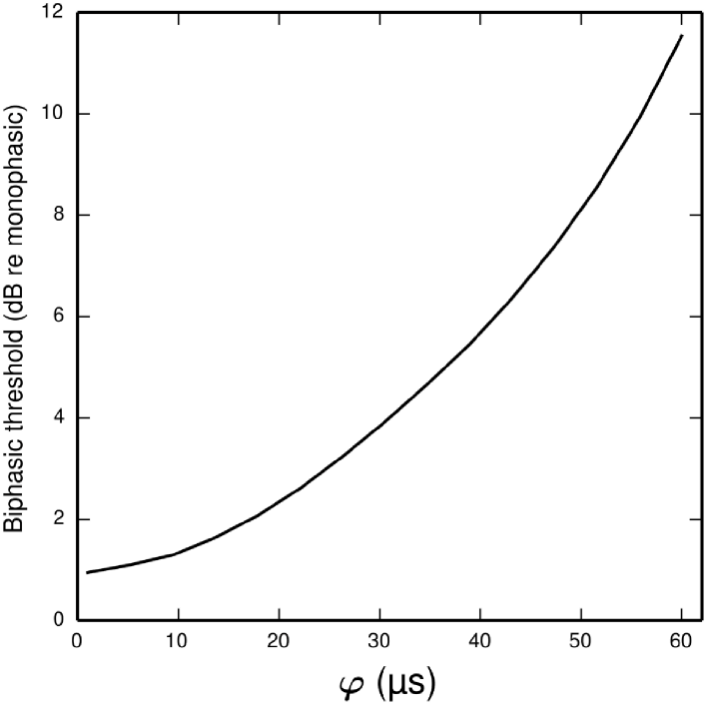
**Plot showing how the threshold of a cathodic-anodic biphasic stimulus depends on the model parameter φ in the BLIF neuron**. The ordinate is given in dB relative to the threshold of a monophasic stimulus of 40 μs duration. The curve is generated for a biphasic stimulus of 40 μs/phase duration and zero IPG.

In the ANF, the threshold of a cathodic-anodic biphasic stimulus is elevated relative to that of an equivalent monophasic stimulus (Shepherd and Javel, 1999; Miller et al., 2001). Increasing the φ parameter of the BLIF neuron increases the thresholds of cathodic-anodic biphasic stimuli, while leaving the thresholds of monophasic stimuli unchanged. Figure 8 quantifies the effect of φ on the biphasic/monophasic threshold difference. To understand why φ affects the threshold of a biphasic stimulus, we note that a spike is canceled if *t*_1_ > *T*_*Q*0_(*t*_0_). The effect of φ is to ensure that *t*_1_ is greater than *t*_0_ + φ, and so increasing φ increases the probability that *t*_1_ > *T*_*Q*0_ (*t*_0_), thus increasing the probability of spike cancelation.

#### Biphasic input-output function

**Figure 9:**
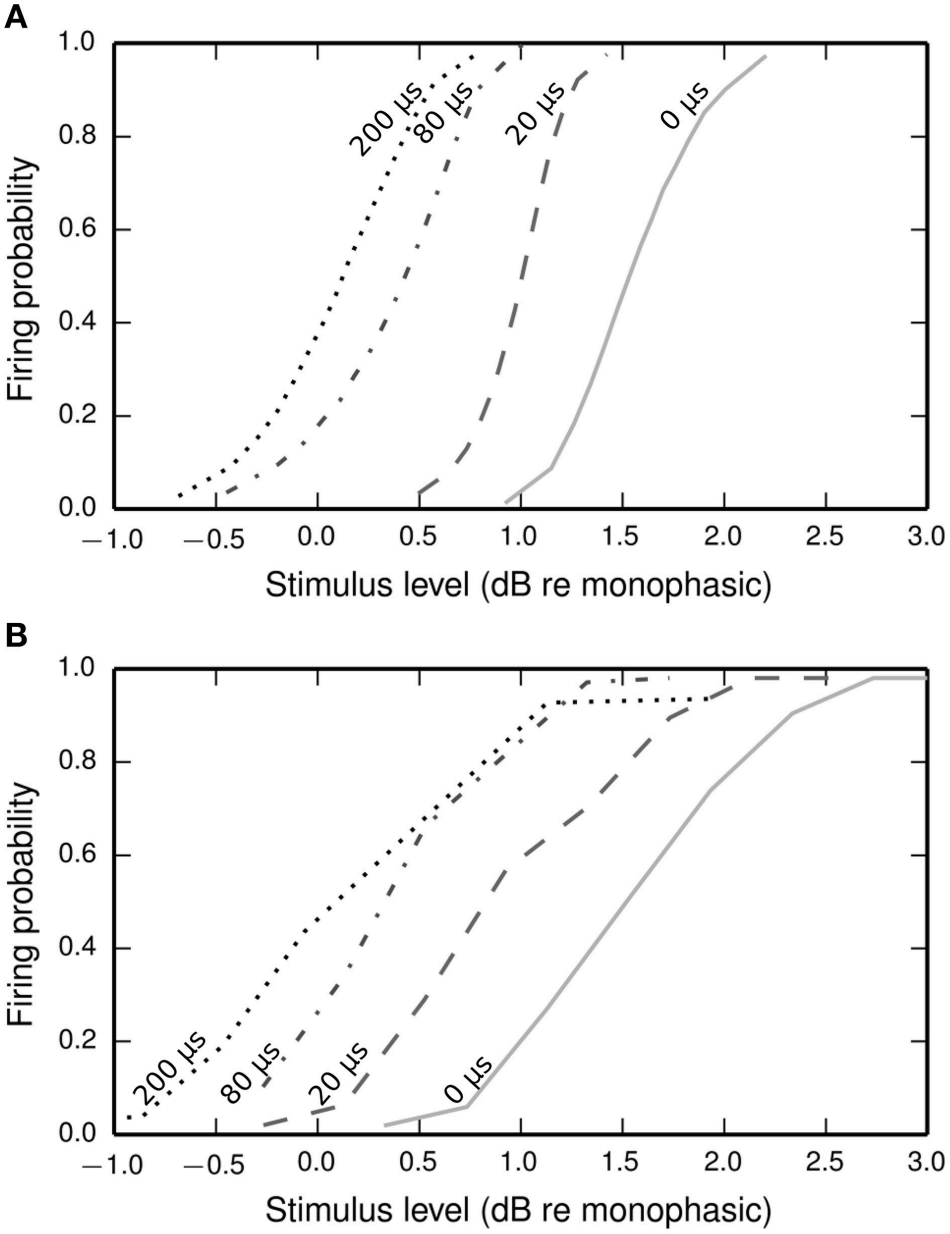
**Input-output functions for the BLIF neuron (A) and a cat ANF (Shepherd and Javel, 1999; B), both stimulated with a cathodic-anodic biphasic stimulus of 100 μs/phase duration at various IPGs (dotted line: 200 μs; dash-dotted line: 80 μs; dashed line: 20 μs; solid line: 0 μs)**. The means of the input-output functions of the cat ANF are well predicted by the BLIF neuron (see Figure 10A), although the slopes are steeper in the BLIF neuron than in the cat ANF (see Figure 10B). Stimulus levels are given in decibels relative to the threshold of a 100 μs duration monophasic stimulus (the threshold for the ANF is projected from the available data; see Figure 10A).

Figure 9 plots input-output functions of the BLIF neuron and a cat ANF (Shepherd and Javel, 1999) in response to a cathodic-anodic biphasic pulse presented at various IPGs. In both the model and the ANF, increasing the IPG of the stimulus has the effect of shifting the entire curve toward lower thresholds. The model parameter φ was chosen so that the curves of the model and the data overlap at the mean when the IPG is 0.

**Figure 10:**
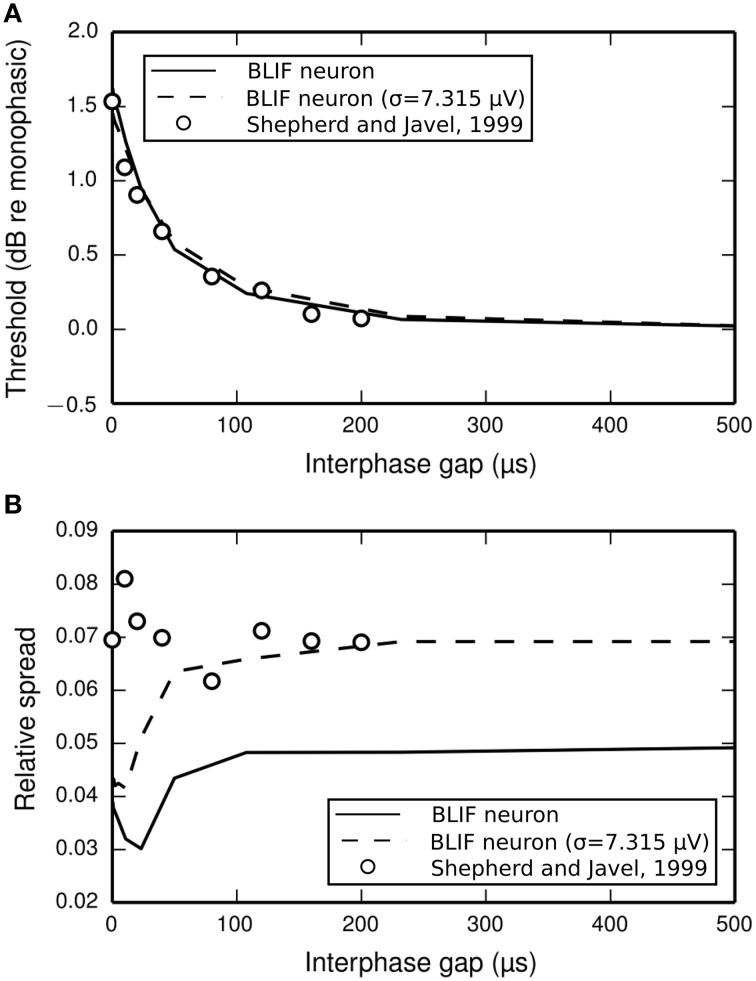
**(A)** Threshold and **(B)** RS of the BLIF neuron (solid lines) and a cat ANF (open circles; Shepherd and Javel, 1999) depend on the IPG of a cathodic-anodic biphasic stimulus (100 μs/phase duration). Also plotted are the results after re-parameterizing the model to have a monophasic RS of 7% (dashed line) by using σ = 7.315μV.

The input-output functions shown in Figure 9 are summarized in Figure 10 by plotting the threshold (Figure 10A) and RS (Figure 10B) with respect to IPG. The thresholds of the ANF are quantitatively predicted by the model at all IPGs. The RS of the model approaches its monophasic value of 5% as the IPG increases beyond ~100 μs. However, the ANF has an average RS of 7%, with no apparent dependence on IPG. This is in part because the monophasic RS of the model was chosen to reproduce data from a different neuron to that being compared presently (see Figure 1A; to our knowledge, no study has been published that contains all the data necessary to parameterize the model using results from only a single neuron). To show that the BLIF neuron can exhibit larger values of RS, it was re-parameterized (by changing σ) to respond to a monophasic stimulus with a RS of 7%. The biphasic input-output functions resulting from this new parameterization are summarized by the dashed lines Figures 10A,B. Changing σ did not affect the monophasic strength-duration function of the model, or the mean latency or jitter of the model's response to a monophasic stimulus. Regardless of the monophasic RS, the model shows a dip in RS at short IPGs (< ~100 μs), a trend that is not present in the ANF. At its peak deviation, the RS of the model is 4/10-ths that of the mean RS of the data.

A caveat in using the data from Shepherd and Javel (1999) is that they were obtained with a bipolar electrode, and thus, there is no clear definition as to which polarity of current is anodic and which is cathodic. The model presented here is a point-neuron model that is excited only by cathodic current. The present data was used due to lack of availability of other monopolar ANF data investigating the effect of the IPG on the threshold of a cathodic-anodic biphasic stimulus. We justify our use of the bipolar data by noting that Shepherd and Javel report that both polarity orders produced similar results with negligible (0.2 dB) changes in threshold. Further, the trends in the data are comparable to those from other studies of single neurons (from animal preparations and computer models) where monopolar, cathodic-anodic biphasic stimulation was used (van den Honert and Mortimer, 1979; Gorman and Mortimer, 1983; Hofmann et al., 2011; Weitz et al., 2011, 2014).

#### Pseudo-monophasic threshold

The phases of a biphasic stimulus do not need to have equal duration for the stimulus to be charge balanced. A pseudo-monophasic stimulus has been proposed for use by cochlear implants (e.g., van Wieringen et al., 2008), where the duration of one phase, typically the second, is extended relative to the other. The amplitude of the extended phase is reduced to maintain charge balance.

**Figure 11:**
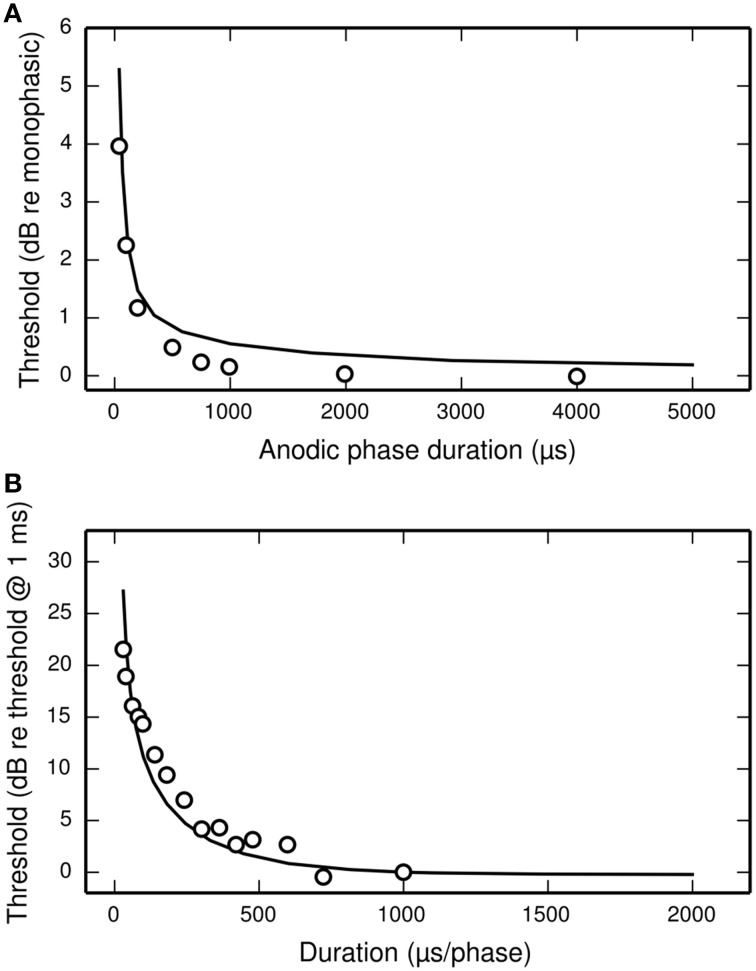
**The dependence of threshold on phase duration under cathodic-anodic biphasic stimulation. (A)** The effect on threshold of increasing the anodic-phase duration in the BLIF neuron (solid line) and an ANF (Miller et al., 2001; open circles). The stimulus is an asymmetric, cathodic-anodic biphasic pulse (40 μs leading-phase duration) of varying anodic-phase duration. The amplitude of the anodic phase is adjusted to maintain charge balance. **(B)** Strength-duration functions of the BLIF neuron (solid line) and a cat ANF (Shepherd et al., 2001; open circles) when stimulated with a symmetric, cathodic-anodic biphasic stimulus (0 μs IPG).

Figure 11A plots the threshold of the BLIF neuron in response to a cathodic-anodic pseudo-monophasic stimulus of varying anodic-phase duration (APD, varying from 40 to 500 μs). The BLIF neuron quantitatively predicts data from a cat ANF (Miller et al., 2001) at short APDs. However, as the APD increases beyond ~200 μs, the BLIF neuron over-predicts threshold by a maximum of 0.47 dB (APD = 748 μs). This over-prediction is small when compared to the 5 dB range in the threshold data. The threshold of the BLIF neuron also returns to its monophasic value, however the convergence becomes much slower than in the ANF once the APD exceeds ~200 μs. At an APD of 5 ms, the BLIF neuron's threshold is 0.24 dB from its monophasic value. It is also worth noting that phase durations longer than a few 100 μs would be very unusual in practice.

#### Biphasic strength-duration function

The threshold of a biphasic stimulus also depends upon its overall duration; increasing its duration—by equally increasing the duration of both its phases—decreases its threshold. Figure 11B plots the strength-duration functions of the BLIF neuron and a cat ANF (Shepherd et al., 2001), each using a cathodic-anodic biphasic pulse as the stimulus. The BLIF neuron predicts the trend that increasing the phase duration decreases the threshold. The BLIF neuron does not precisely predict the data, differing with a root-mean-square deviation of 2.5 dB. This value is small in comparison to the range in the threshold data (21.5 dB).

#### Temporal response statistics

**Figure 12:**
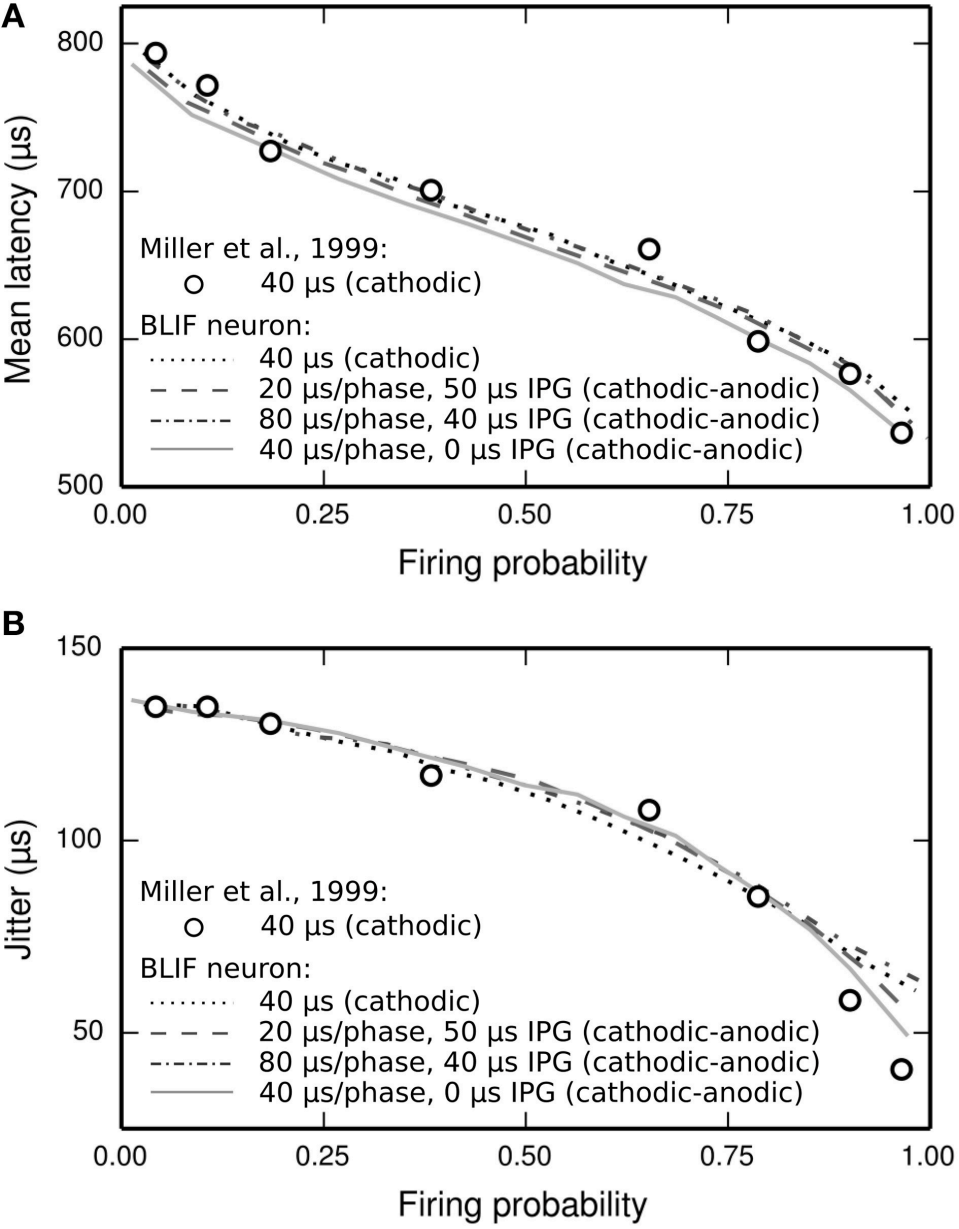
**The temporal response statistics of the BLIF neuron are well predicted by firing probability, regardless of the stimulus pulse shape. (A)** Mean latency and **(B)** jitter of the BLIF neuron's response to various configurations of a cathodic-anodic stimulus (solid lines: phase duration: 40 μs/phase, IPG: 0 μs; dashed lines: phase duration: 20 μs/phase, IPG: 50 μs; dash-dotted lines: phase duration: 80 μs/phase, IPG: 40 μs) and a monophasic stimulus (40 μs duration; dotted lines). Also plotted are the monophasic data with which the model was parameterized (open circles; 40 μs pulse duration; Miller et al., 1999).

The latency distribution of the ANF's response to a biphasic stimulus depends on stimulus level, with higher levels incurring less jitter (Javel and Shepherd, 2000). The underlying assumption of the TLIF neuron is that the latency distribution of the response is well predicted by the firing probability alone, regardless of the shape of the current pulse waveform (the validity of this assumption is discussed in Section Latency Distribution's Dependence on Firing Probability). In Figure 12, we show that the temporal statistics (mean latency, Figure 12A, and jitter, Figure 12B) of the BLIF neuron's response to a monophasic stimulus and cathodic-anodic biphasic stimuli of arbitrary shape are well predicted by firing probability, consistent with the assumption of the TLIF neuron.

### Summary

The BLIF neuron responds to a cathodic-anodic biphasic stimulus with an increased threshold relative to that of a monophasic stimulus. The extent of this increase in threshold can be controlled by setting the φ parameter of the model. The effect of φ on the monophasic response statistics is negligible.

The BLIF neuron quantitatively predicts the thresholds of a cat ANF responding to a biphasic stimulus across a range of IPGs between 0 and 200 μs. The BLIF neuron correctly predicts that the threshold approaches its asymptote as the IPG extends beyond ~200 μs. Also predicted is the effect on threshold of increasing the duration of both phases of a cathodic-anodic biphasic stimulus. Further, the effect on threshold of only increasing the duration of the second phase (while adjusting the amplitude of the second phase to maintain charge balance) is also predicted at durations below ~200 μs.

Consistent with the assumptions of the TLIF neuron, the latency distribution of the BLIF neuron's response to a stimulus is well predicted by the probability of the stimulus evoking a spike, regardless of its shape. If the stimulus is monophasic, then the BLIF neuron maintains the input-output and strength-duration functions of the SLIF neuron.

## Discussion

In this paper, we extended the SLIF neuron so as to reproduce the temporal response statistics of the ANF's response to a monophasic stimulus, resulting in the TLIF neuron. We then further extended the TLIF neuron to realistically reproduce how the threshold of the response to a monophasic stimulus is affected by the introduction of a trailing, anodic phase. This resulted in the BLIF neuron. An important property of both the TLIF and the BLIF neuron is that they do not affect the fitted response statistics of the models that they extend. Thus, the TLIF neuron maintains the input-output and strength duration functions of the SLIF neuron, and the BLIF neuron responds to a monophasic stimulus with the same latency distribution and firing probability as the TLIF neuron. Further, the response of the BLIF neuron to a biphasic stimulus has the same latency distribution as that of the TLIF neuron's response to a monophasic stimulus of equal firing probability. These properties of the TLIF and BLIF neurons makes them simple to parameterize so as to reproduce ANF data.

### Latency distribution's dependence on firing probability

The assumption underlying both the TLIF and BLIF neurons is that the mean latency distribution of the response to a stimulus is well predicted by probability of that stimulus evoking a response. If the assumption is correct, then the latency and jitter of the responses to two distinct stimuli should be the same, provided the stimuli are all presented at threshold level. We assess the validity of the assumption by comparing the mean latencies and jitters of the responses of cat ANFs stimulated by monophasic and biphasic stimuli presented at threshold level, using data collected by Miller et al. (2001). The mean latency of the responses to the biphasic stimulus was 88% that of the responses to the monophasic stimulus. The difference in jitter of the responses to the monophasic and biphasic stimuli was not statistically significant. The difference in threshold between the monophasic and biphasic stimuli was 4 dB, or 3.5 times the monophasic dynamic range (measured as the increase in stimulus level required to increase the probability of firing from 10 to 90%). While the assumption mispredicts the mean latency data by 12%, it correctly predicts the jitter data.

### Comparison to previous models

The TLIF and BLIF neurons are extensions to the well-studied leaky integrate and fire (LIF) neuron, which has formed the basis of many models of the electrically stimulated ANF (Stocks et al., 2002; Hamacher, 2004; Chen and Zhang, 2007; Chen, 2012; Goldwyn et al., 2012). Of these models, only the Hamacher model reproduces the temporal response statistics (mean latency and jitter) of the auditory nerve fiber, and their dependence on stimulus level. However, in the Hamacher model, the stimulus is half-wave rectified (Fredelake and Hohmann, 2012), and thus, it cannot reproduce the dependence of the threshold or the temporal response statistics on the IPG and IPD.

The TLIF neuron bears similarities to the Hamacher model. Both models use the probability of a spike being emitted to derive a latency distribution with which to respond. However, the TLIF neuron and the Hamacher model differ in how they predict the probability of the stimulus evoking a response. The TLIF neuron uses the probability of the membrane potential exceeding threshold at any time during the action potential initiation period. The Hamacher model, however, uses the membrane potential at the moment the excitatory phase of the stimulus ceases. This requires knowledge of the time of cessation, and so affects its ability to respond to more complex stimuli, for example a pulse train where each individual pulse is of a level insufficient to evoke a response in isolation. Due to temporal facilitation, the pulse train will evoke a response with high probability. In such a case, the time of stimulus cessation is no longer clearly defined. Further, pulses occurring during the action potential initiation period will bring forward the time of spiking while simultaneously reducing its temporal variability (supporting evidence: Figure 6; Heffer et al., 2010). For this reason, we were motivated to develop a model which would not require direct information about the stimulus (such as the time of excitatory-phase cessation), and which would allow the timing of the response to be continuously affected by stimulation after threshold crossing, during the action potential initiation period.

The model by Goldwyn et al. (2012) is a point-process model that processes the membrane potential of the leaky integrate and fire (LIF) neuron with a non-linearity to introduce stochasticity to its input-output function. The resulting signal can be interpreted as the probability density function of the time of spiking, and is processed by a linear filter to introduce temporal stochasticity. The model parameters are functions of the time since last spiking, allowing refractory effects to be modeled. While Goldwyn et al. only parameterize the jitter for a stimulus presented at threshold level, they find empirically that the stimulus level affects the jitter of the model similarly to the jitter of the ANF. However, the mean latency of the ANF's response, and its dependence on stimulus level, is not modeled.

The model of Goldwyn et al. may be parameterized to quantitatively reproduce the response statistics of a range of biphasic stimulus shapes. However, we found that once fitted to a cathodic-anodic biphasic stimulus, increasing the IPG above ~30 μs had very little effect on the threshold of the model. This is in contrast to the ANF, which is sensitive to changes in IPG up to ~250 μs. The reason for the Goldwyn et al. model's rapid convergence to monophasic threshold is the same as the reason for the SLIF neuron's rapid convergence: the decision as to whether or not a spike will be emitted occurs before the trailing phase of the stimulus can have an effect on the firing probability. It is in this respect that the BLIF neuron differs from the previous phenomenological models that we are aware of: the BLIF neuron introduces the possibility of spike cancelation after spike initiation, allowing the trailing phase of a cathodic-anodic biphasic stimulus to continue to affect the firing probability of the response for much longer durations.

### Spike cancelation via current integration

The BLIF neuron is based on the assumption that a delay exists between the initial depolarization of the ANF and the moment at which the action potential is generated. During this delay, it is assumed that the action potential may be canceled by anodic current. By this means, the biphasic threshold is increased relative to the monophasic threshold. This assumption is consistent with a study by van den Honert and Mortimer (1979), who propose that there exists a delay, the vulnerable period, between the depolarization of the neural membrane and the opening of the sodium ion channels. During the vulnerable period, the activation of the sodium ion channels may be prevented by anodic current, resulting in the abolishment of the action potential. This view is later reaffirmed with reference to the cat ANF by Miller et al. (2001), who propose that the continued integration of the current during the vulnerable period results in the increased threshold of biphasic stimuli. This is consistent with the BLIF neuron integrating the stimulus current during the action potential initiation period and canceling the spike if the net charge is negative.

There is one aspect of this mechanism which it not consistent with the empirical data. Whilst RS does not vary with pulse duration for monophasic stimuli (Verveen and Derksen, 1965), it does for biphasic stimuli (Bruce et al., 1999). The BLIF inherits this aspect of the response from the TLIF model. This means that the model will fail to predict any variation in dynamic range afforded by varying pulse duration. Although it is unclear whether this would seriously limit the evaluation of different stimulation strategies, this limitation should be borne in mind. We note that RS varies relatively little over phase durations typically used in CIs, and below a few 100 μs. It is not clear how the model could be modified to reproduce this. It may indicate that the linear integration of current for the cancelation mechanism is not correct. Nor is it clear why this is observed in the data. Bruce et al. speculated that this could be due to the level of noise at the initial site increasing with pulse width, or that the site of action-potential initiation depends on pulse width in a way that does not occur with monophasic stimuli.

### Effect of order on biphasic stimulation

It has previously been observed that the ANF responds with similar threshold levels to biphasic stimuli, regardless of the order of the opposite-polarity phases (Macherey et al., 2006), and that the threshold of a biphasic stimulus depends on IPG similarly, regardless of the phase order (Shepherd and Javel, 1999). This suggests that excitation by a biphasic stimulus occurs similarly, regardless of the phase order. Supporting evidence for this hypothesis can be found in Carlyon et al. (2005), where a filter-based model is used to predict human thresholds for stimuli of varying IPG. The model predicts the same threshold for a train of cathodic-anodic pulses as it does for a train of anodic-cathodic pulses.

The BLIF neuron is formed around the hypothesis that spike cancelation is responsible for the increased threshold of cathodic-anodic biphasic stimuli. In the BLIF neuron, this hypothesis is fundamentally only applicable if the excitatory, cathodic, phase is leading, and so appears to be at odds with the results from the literature. Here we outline how the hypothesis behind the BLIF neuron may be consistent with the results from the literature.

In an idealized axon, cathodic currents depolarize the nodes of Ranvier proximal to the electrode, whereas anodic current depolarize those distal to the electrode (Ranck, 1975; Rattay, 1986). Depolarization due to cathodic current is more efficient than depolarization due to anodic current. With this in mind, a cathodic-anodic biphasic stimulus will depolarize-then-hyperpolarize the nodes of Ranvier proximal to the electrode, whereas an anodic-cathodic biphasic stimulus will depolarize-then-hyperpolarize the nodes of Ranvier distal to the electrode—but with less efficiency. Therefore, it could be the case that a biphasic stimulus always excites the neuron by first depolarizing the excited node of Ranvier (which may be distal or proximal to the electrode), regardless of the order of the opposite-polarity phases. If this is the case, the threshold differences between cathodic-anodic and anodic-cathodic biphasic stimuli should be well predicted by the threshold differences between cathodic and anodic monophasic stimuli.

### Pulse train response

This paper only considers the response of the neuron to a single, brief, stimulus pulse. To be useful, the model must be capable of responding to trains of pulses, such as those generated by cochlear implants. There is no limiting factor preventing the model from responding with a train of spikes. The model makes no assumptions about the pulse shape, and after spiking or spike cancelation, the model can be reset. The threshold of the model can be elevated after spiking to emulate the relative refractory period using a method similar to that described by Goldwyn et al. (2012) and Hamacher (2004).

## Author contributions

CH designed, implemented the model, obtained all results from the model, and wrote the manuscript. CS and BS supervised the work, contributed conceptually to the design of the model, and the direction of the work, and helped to write the manuscript.

## Funding

This work was funded by the Medical Research Council (U135097127 and U135097132). BS was also supported by the Bernstein Center for Computational Neuroscience, BMBF 01 GQ 1004B.

### Conflict of interest statement

The authors declare that the research was conducted in the absence of any commercial or financial relationships that could be construed as a potential conflict of interest.
